# Regulation of B cell differentiation by the ubiquitin-binding protein TAX1BP1

**DOI:** 10.1038/srep31266

**Published:** 2016-08-12

**Authors:** Nobuko Matsushita, Midori Suzuki, Emi Ikebe, Shun Nagashima, Ryoko Inatome, Kenichi Asano, Masato Tanaka, Masayuki Matsushita, Eisaku Kondo, Hidekatsu Iha, Shigeru Yanagi

**Affiliations:** 1 Laboratory of Molecular Biochemistry, School of Life Sciences, Tokyo University of Pharmacy and Life Sciences, Hachioji, Tokyo, 192-0392, Japan; 2 Department of Microbiology, Oita University Faculty of Medicine, Yufu, Oita, 879-5593, Japan; 3 Laboratory of Immune Regulation, School of Life Sciences, Tokyo University of Pharmacy and Life Sciences, Hachioji, Tokyo, 192-0392, Japan; 4 Department of Molecular and Cellular Physiology, Graduate School of Medicine, University of the Ryukyus, Okinawa, 903-0215, Japan; 5 Division of Molecular and Cellular Pathology, Niigata University Graduate School of Medical and Dental Sciences, Niigata, 951-8510, Japan

## Abstract

Tax1-binding protein 1 (TAX1BP1) is a ubiquitin-binding protein that restricts nuclear factor-κB (NF-κB) activation and facilitates the termination of aberrant inflammation. However, its roles in B-cell activation and differentiation are poorly understood. To evaluate the function of TAX1BP1 in B cells, we established TAX1BP1-deficient DT40 B cells that are hyper-responsive to CD40-induced extracellular signal-regulated kinase (ERK) activation signaling, exhibit prolonged and exaggerated ERK phosphorylation and show enhanced B lymphocyte-induced maturation protein 1 (Blimp-1; a transcription factor inducing plasma cell differentiation) expression that is ERK-dependent. Furthermore, TAX1BP1-deficient cells exhibit significantly decreased surface IgM expression and increased IgM secretion. Moreover, TAX1BP1-deficient mice display reduced germinal center formation and antigen-specific antibody production. These findings show that TAX1BP1 restricts ERK activation and Blimp-1 expression and regulates germinal center formation.

B cells play a critical role in immune responses to invasive pathogens. When they encounter an exogenous antigen, naive B cells are activated and form germinal centers (GCs) in the follicles of peripheral lymphoid tissues. These GCs are composed of a dark zone, wherein B-cell division and somatic hypermutation (SHM) primarily occur, and a light zone, wherein B cells undergo selection depending on the affinity of their B-cell receptors toward the antigen^1^,^2^,^3^. After proliferation and SHM in the dark zone, B cells move to the light zone, followed by re-entry into the dark zone or exit from the GC as differentiated memory B cells and plasma cells.

The fate of an activated B cell is determined by signals from its receptors and other GC cells, including T cells and dendritic cells. These signals regulate multiple modulators and transcription factors that affect GC B cell responses^4^,^5^, and accordingly the expression of transcriptional factors in this network is strictly regulated and cross-modulated. The transcription factors B-cell lymphoma 6 (Bcl-6) and paired box gene 5 (Pax5) are highly expressed during B-cell initiation and proliferation in the GC^6^,^7^. However, the expression of these transcription factors is restricted in plasma cells, and the transcription factors B lymphocyte-induced maturation protein 1 (Blimp-1), interferon regulatory factor 4 (IRF-4), and X-box binding protein 1 (XBP-1) are induced in B cells involved in plasma cell differentiation^8^. Notably, Blimp-1 represses the transcription of Bcl-6, whereas Bcl-6 inhibits the transcription of Blimp-1^9^.

Although mutual relationships between transcription factors associated with GC have been clarified, the signals that regulate the expression of these transcription factors remain unknown. GC B cells are activated by stimuli through several receptors, including B cell receptors (BCRs), CD40, a member of the tumor necrosis factor (TNF) receptor family and toll-like receptors (TLRs). Subsequently, the resulting signals are transduced through several different pathways, wherein lysine K63 (K63)-linked polyubiquitination is an important regulatory mechanism for protein–protein interactions triggering the nuclear factor-κB (NF-κB) and mitogen-activated protein kinase (MAPK) pathways^10^,^11^,^12^.

Tax1-binding protein 1 (TAX1BP1) was initially identified as a human T-cell leukemia virus type 1 Tax-binding protein^13^. TAX1BP1 functions as a ubiquitin-binding adaptor protein for the TNFα -inducible gene 3 (Tnfaip3)-encoded ubiquitin-modifying enzyme A20, which is composed of deubiquitinase and E3 ligase domains and inactivates K63-linked polyubiquitinated receptor-interacting protein kinase 1 (RIP1) and tumor necrosis factor receptor-associated factor 6 (TRAF6)^14^,^15^. The complex formed by A20 and its regulatory molecule TAX1BP1 acts as a central negative regulator in multiple NF-κB-activating signaling pathways by cleaving K63-linked polyubiquitin chains and conjugating K48-linked polyubiquitin chains to its substrate, thereby inducing protein degradation^16^. In mice, targeting of TAX1BP1 causes hyperinflammations including inflammatory cardiac valvulitis and skin dermatitis through NF-κB dysregulation^15^,^17^. Cultured TAX1BP1-deficient cells are more hypersensitive to TNFα and IL-1β and exhibit increased NF-κB activation compared with wild-type (WT) cells. A20-deficient (*Tnfaip3*^*−/−*^) mice exhibit severe inflammation and cachexia and die prematurely. A20-deficient cells are hypersensitive to both lipopolysaccharide and TNFα stimulus and fail to terminate NF-κB responses. To regulate B-cell responses, A20 restricts CD40-induced NF-κB signals that repress Fas-mediated cell death, and B-cell-specific A20-deficient mice display elevated germinal center B-cell numbers and autoantibody production^18^,^19^. These data suggest that TAX1BP1 may play a role in B-cell differentiation; however, the role of TAX1BP1 in regulating B-cell responses remains unknown.

In this study, we generated TAX1BP1-deficient DT40 B cell lines to address the role of TAX1BP1 in B cells. The chicken B-cell line DT40 expresses surface IgM, but it continues Ig gene conversion with apparent arrest at the bursal B cell stage^20^. TAX1BP1-deficient DT40 cells exhibited a plasmacytic phenotype with impaired cell surface IgM expression, significantly enhanced ERK phosphorylation, and increased expression of the plasma cell transcription factors Blimp1, IRF4, and XBP1. In mice, targeting of TAX1BP1 led to impaired GC B cells and GC formation and a subsequent decrease in antigen-specific antibody production. These results demonstrate that TAX1BP1 is required for B-cell differentiation and GC formation.

## Results

### Targeted disruption of TAX1BP1 in DT40 cells

**Figure 1:**
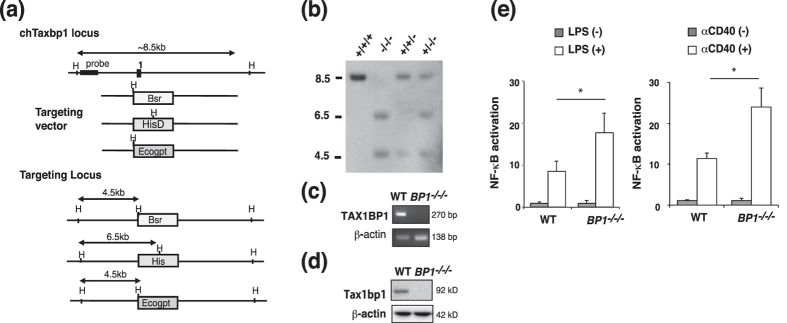
Generation of a TAX1BP1-deficient chicken DT40 B cell line. (**a**) We disrupted three alleles of the *Tax1bp1* gene in the chicken B cell line DT40 via sequential transfection with the targeting vectors TAX1BP1-bsr, -His, and -Ecogpt. (**b**) Target integration was monitored by Southern blotting of HindIII-digested genomic DNA. (**c**) Reverse transcriptase-PCR was used to analyze TAX1BP1 expression in WT and TAXBP1^*−/−/−*^cells. (**d**) Immunoblot with TAX1BP1 antibody. (**e**) TAX1BP1 inhibits LPS- and anti-CD40 antibody (αCD40)-induced NF-κB activation in B cells. TAX1BP1-deficient DT40 cells (*BP1*^*−/−/−*^) exhibited higher NF-κB activity in response to lipopolysaccharide (LPS) and αCD40 compared with WT cells. Cells were transfected with an NF-κB-luc reporter and a renilla reporter pRL-CMV plasmid as an internal control. At 24 h after transfection, the indicated concentrations of αCD40 (2.5 μg/ml) and LPS (50 ng/ml) were added for 8–24 h, after which the cells were harvested and subjected to a luciferase assay. Relative NF-κB activation is shown in comparison to the activation level in unstimulated WT cells (arbitrarily set at 1). The results are shown as the means and standard errors of the mean (s.e.m.; n = 3). *P < 0.05, P values are based on two-tailed Student’s t tests.

To identify the role of TAX1BP1 in B cells, we generated TAX1BP1 knockout cells in the chicken B-cell line DT40. The chicken *Tax1bp1* gene is located on chromosome 2, which is trisomic in DT40 cells. We generated deletion constructs comprising different marker genes (*bsr*, *his*, and *ecogpt*), integration of which into the first exon of the *Tax1bp1* allele resulted in a deletion of the coding base pairs 1–179 as previously described^21^ (Fig. 1a). *Tax1bp1* gene disruption was verified by Southern blot analysis using the indicated 5′ probe (Fig. 1b). Reverse transcription PCR analysis confirmed that *TAX1BP1*^*−/−/−*^ DT40 cells did not express TAX1BP1 transcripts (Fig. 1c). In addition, we confirmed the expression of TAX1BP1 protein in WT DT40 cells but not in *TAX1BP*^*−/−/−*^ cells using a specific TAX1BP1 antibody (Fig. 1d). To examine the functional effects of TAX1BP1 on NF-κB activation in B cells, we measured transcriptional activity using an NF-κB-responsive reporter. Disruption of TAX1BP1 enhanced both LPS and anti-CD40 antibody (αCD40)-mediated NF-κB activation compared with WT DT40 cells (Fig. 1e).

### TAX1BP1 restricts ERK activation in B cells

**Figure 2:**
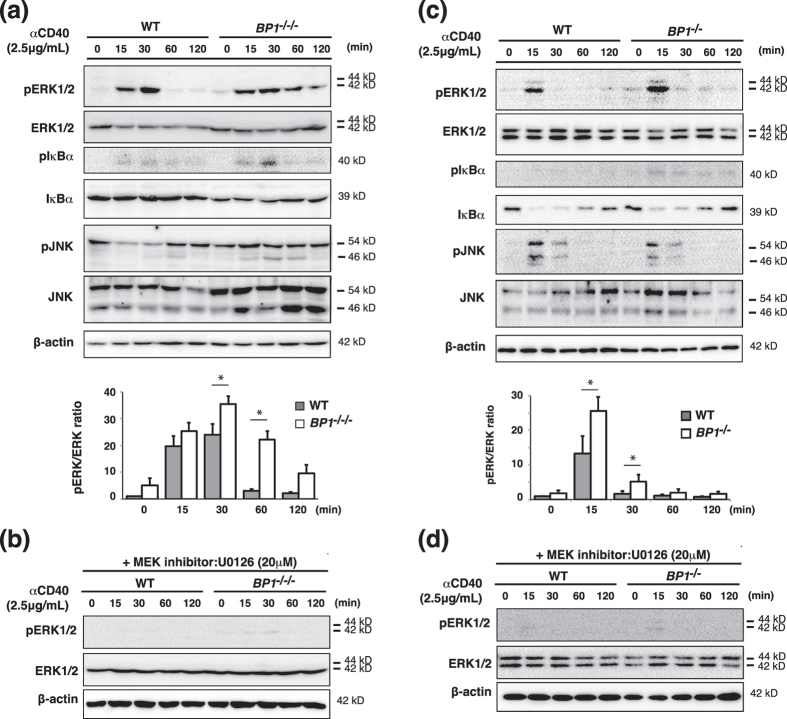
Disruption of TAX1BP1 in the DT40 B cell line enhanced ERK pathway activation in response to CD40 stimulation. Western blot of whole cell lysates stimulated for the indicated time points with anti-CD40 antibody (αCD40). The results are representative immunoblot analyses from three independent experiments. The amount of ERK protein phosphorylation was determined as the ratio of phospho-ERK protein to total ERK protein and normalized with respect to unstimulated WT cells. The results are shown as the means and standard deviation (n = 3). Statistical analysis was performed by two-tailed Student’s t tests (*P < 0.05 compared with WT samples). (**a**) Whole cell lysates were prepared from αCD40 stimulated WT DT40 cells (WT) and TAX1BP1-deficient DT40 cells (*BP1*^*−/−/−*^). (**b**) The MEK inhibitor U0126 reduced ERK pathway activation in TAX1BP1-deficient cells. Shown is a western blot of whole cell lysates stimulated for the indicated time points with αCD40 in the presence of U0126 (20 μM). (**c**) Whole cell lysates were prepared from αCD40 stimulated splenic B cells from WT and TAX1BP1-deficient mice (*BP1*^*−/−*^). (**d**) The MEK inhibitor U0126 reduced ERK pathway activation in splenic B cells from TAX1BP1-deficient mice (*BP1*^*−/−*^). Shown is a western blot of whole cell lysates stimulated for the indicated time points with αCD40 in the presence of U0126 (20 μM).

B cells are regulated by several stimuli through various receptors such as BCR, TLR, and CD40. Signaling through CD40, a TNF receptor family member, induces NF-κB activation, resulting in B cell activation and survival^22^. TAX1BP1 has been shown to interact with A20 to form a ubiquitin-editing complex that functions as a negative regulator for NF-κB activation via TRAF6 and RIP1 degradation^14^,^23^. A20-deficient B cells display enhanced CD40-induced canonical NF-κB activation, evidenced by increased inhibitor of kappa B (I-κB) phosphorylation and degradation, but not MAPK pathway activation^18^. To examine whether TAX1BP1 similarly restricts CD40-induced NF-κB signals, we stimulated WT and *TAX1BP1*^*−/−/−*^ DT40 cells with αCD40 for the indicated time intervals. TAX1BP1-deficient B cells exhibited slightly enhanced I-κB phosphorylation (Fig. 2a). Next we examined the role of TAX1BP1 in the activation of MAPKs, including JNK and ERK. We detected significantly elevated and prolonged ERK phosphorylation in *TAX1BP1*^*−/−/−*^ DT40 cells. The ERK phosphorylation level was calculated as the ratio of phospho-ERK to total ERK protein and was normalized with respect to unstimulated WT cells (Fig. 2a). In addition, the mitogen-activated protein kinase kinase (MEK) inhibitor U0126 reduced ERK phosphorylation in TAX1BP1-deficient cells (Fig. 2b). Taken together, TAX1BP1 deficiency in DT40 cells selectively enhances activation of the ERK pathway in response to αCD40. To confirm the physiological role of TAX1BP1 in mouse B lymphocyte activation, we isolated splenic B cells from TAX1BP1-deficient (*TAX1BP1*^*−/−*^)^15^ and WT mice and measured activation of the NF-κB and ERK pathway. Figure 2c shows that ERK phosphorylation was significantly enhanced in αCD40-stimulated cells, and the MEK inhibitor U0126 reduced ERK phosphorylation in B cells from *TAX1BP1*^*−/−*^ mice (Fig. 2d). Previous studies have shown that TRAF6 contributes to the CD40-mediated activation of ERK and cell proliferation in lymphoid cells and tumor cells^24^,^25^. Following CD40 activation, the K63-specific ubiquitin ligase activity of TRAF6 is rapidly stimulated, leading to TRAF6 autoubiquitination. These resulting polyubiquitin chains may further stabilize the respective signaling complexes and induce ERK activation. We therefore hypothesized that TAX1BP1 inhibits TRAF6 polyubiquitination in these signals and restricts ERK activity. As expected, upon αCD40 stimulation TRAF6 K63-linked polyubiquitination was enhanced in TAX1BP1-deficient cells compared to WT DT40 cells (Supplementary Fig. S1).

### TAX1BP1 negatively regulates expression of the gene encoding Blimp-1 and B cell differentiation

**Figure 3:**
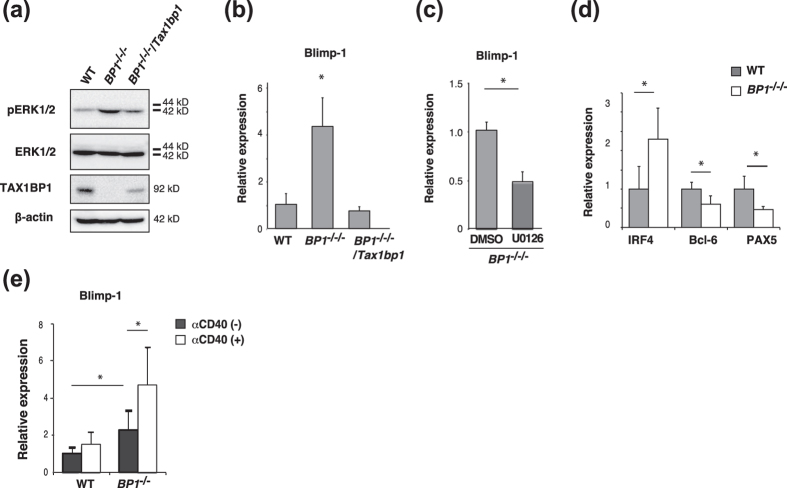
TAX1BP1 restricts expression of the gene encoding Blimp-1 by regulating the ERK pathway. (**a**) Western blot of whole-cell lysates from unstimulated WT, *TAX1BP1*^*−/−/−*^ (*BP1*^*−/−/−*^) and *TAX1BP1*^*−/−/−*^*/Tax1bp1* (*BP1*^*−/−/−*^*/Tax1bp1*; TAX1BP1 defect complemented with TAX1BP1) cell lines. The results are representative of 3 independent experiments. (**b**) Relative quantification of the expression of Blimp-1 transcripts in WT, *BP1*^*−/−/−*^ and *BP1*^*−/−/−*^*/Tax1bp1* cell lines. The Blimp-1 transcript level is shown relative to the level in WT cells, which is arbitrarily set at 1. The results are shown as the means and s.e.m. (n = 4). Statistical analysis was performed by one-way ANOVA (*P < 0.05 compared with WT samples). (**c**) Effect of the MEK inhibitor U0126 (20 μM) on relative Blimp-1 transcript expression in the *BP1*^*−/−/−*^ cell line. The Blimp-1 transcript level is shown relative to the transcript level in *BP1*^*−/−/−*^ cells treated with DMSO, which is arbitrarily set at 1. The results are shown as the means and s.e.m. (n = 4). *P < 0.05. P values are based on two-tailed Student t tests. (**d**) Relative quantification of the levels of IRF4, Bcl-6, and PAX5 transcripts in WT and *BP1*^*−/−/−*^ cell lines. The transcript levels are shown relative to the transcript levels in WT cells, which are arbitrarily set at 1. The results are shown as the means and s.e.m. (n = 4). *P < 0.05. P values are based on two-tailed Student t tests. (**e**) Relative quantification of the levels of Blimp-1 transcripts in isolated splenic B cells, from WT or *TAX1BP1*^*−/−*^ mice (*BP1*^*−/−*^), with or without CD40 activation (2.5 μg/ml, 8 h). The transcript levels are relative to the transcript level in unstimulated splenic B cells from WT mice, which is set at 1. The results are shown as the means and s.e.m. (n = 5). *P < 0.05. P values are based on two-tailed Student t tests.

We restored TAX1BP1 expression in *TAX1BP1*^*−/−/−*^ cells (*TAX1BP1*^*−/−/−*^*/Tax1bp1*) to provide functional complementation and verified the disruption of TAX1BP1-enhanced ERK phosphorylation in B cells and restoration of TAX1BP1-mediated attenuation of ERK activity (Fig. 3a). The ERK signaling pathway is essential for the differentiation of B cells into antibody-secreting plasma cells because it induces the expression of *Prdm1*, which encodes the plasma cell master regulator Blimp-1^26^. According to quantitative PCR analysis, TAX1BP1 deficiency significantly enhanced the expression of Blimp1 compared with WT cells, and the restoration of TAX1BP1 expression restored Blimp-1 suppression (Fig. 3b). The MEK inhibitor U0126, previously shown to reduce ERK phosphorylation, also suppressed Blimp-1 expression in TAX1BP1-deficient cells (Fig. 3c). In addition, TAX1BP1-deficient cells exhibited reduced expression levels of Pax5 and Bcl-6, which are repressed by Blimp-1, whereas the expression of IRF4, which is induced by the NF-κB pathway, was significantly enhanced compared with WT cells (Fig. 3d). To examine the physiological role of TAX1BP1 in expression of Blimp1, we isolated splenic B cells from WT and *TAX1BP1*^*−/−*^ mice and measured Blimp1 expression. Even without CD40 activation, TAX1BP1-deficient splenic B cells showed elevated expression of Blimp1, and after CD40 activation, Blimp1 expression was significantly enhanced compared to WT splenic B cells (Fig. 3e).

### TAX1BP1 deficiency induces aberrant plasma cell differentiation

**Figure 4:**
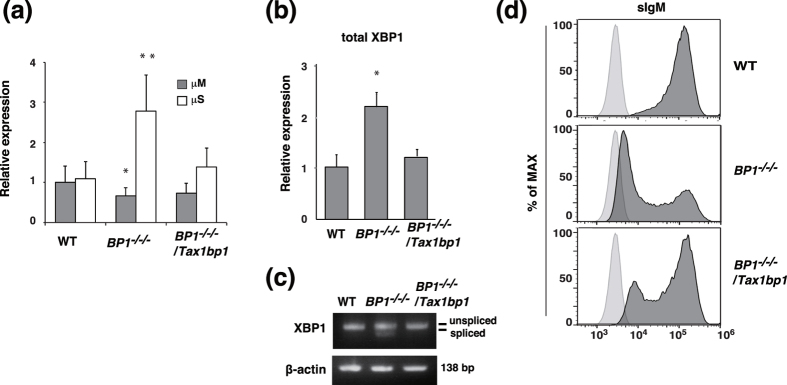
TAX1BP1 deficiency enhances immunoglobulin secretion. (**a**) Relative quantification of transcripts for the membrane (μM) and secretory forms (μS) of the immunoglobulin μ heavy chain in WT DT40 (WT), *TAX1BP1*^*−/−/−*^ (*BP1*^*−/−/−*^) and *TAX1BP1*^*−/−/−*^*/Tax1bp1* (*BP1*^*−/−/−*^*/Tax1bp1*) clones using quantitative PCR analysis. The transcript levels are shown relative to the transcript level of μM in WT cells, which is set at 1. The results are shown as the means and s.e.m. (n = 4). Statistical analysis was performed by one-way ANOVA (**P < 0.01, *P < 0.05 compared with WT samples). (**b**) Quantitative PCR analysis of XBP1 in WT, *BP1*^*−/−/−*^, and *BP1*^*−/−/−*^*/Tax1bp1* cell lines. The transcript levels are relative to the transcript levels in WT cells, which are set at 1. The results are shown as the means and s.e.m. (n = 4). Statistical analysis was performed by one-way ANOVA (*P < 0.05 compared with WT samples). (**c**) RT-PCR analysis of XBP-1 splicing in WT, *BP1*^*−/−/−*^, and *BP1*^*−/−/−*^*/Tax1bp1* cell lines. The results are representative of 3 independent experiments. (**d**) Surface immunoglobulin (sIgM) expression in WT, *BP1*^*−/−/−*^, and *BP1*^*−/−/−*^*/Tax1bp1* cell lines was analyzed by flow cytometry. Histograms indicate the relative cell number and logarithmic fluorescence intensity.

To examine how the aberrant expression of Blimp1 accelerates the process of antibody-secreting plasma cell differentiation in *TAX1BP1*^*−/−/−*^ cells, we analyzed IgM secretion by measuring secretory type IgM heavy chain (μS) transcript expression. The relative number of secretory type IgM heavy chain (μS) transcripts was significantly increased in *TAX1BP1*^*−/−/−*^cells compared with WT cells. In contrast, the expression level of transcripts encoding membrane-type IgM heavy chain (μM) was decreased compared with WT cells. Restoration of TAX1BP1 expression downregulated the expression of μS, but not μM, transcripts in *TAX1BP1*^*−/−/−*^*/Tax1bp1* cells (Fig. 4a). To determine the IgM-secreting ability of TAX1BP1-deficient DT40 cells, ELISA assays were performed on culture medium from WT, *TAX1BP1*^*−/−/−*^ and *TAX1BP1*^*−/−/−*^*/Tax1bp1* cells. Although IgM secreted by WT cells was nearly undetectable, *TAX1BP1*^*−/−/−*^cells secreted IgM in culture medium, and the reintroduction of TAX1BP1 reduced IgM secretion (Supplemental Fig. S2a). Antibody-secreting plasma cells feature a constitutively active unfolded protein response (UPR) for which the transcription factor XBP1 is required. During plasma cell differentiation from stimulated B cells, active UPR signaling leads to the enzymatic splicing of XBP1 mRNA, thereby enabling expression of the transcription factor XBP1 and inducing cellular structural changes to facilitate high antibody production rates^27^,^28^. According to quantitative PCR analysis, total XBP1 mRNA expression was significantly increased in *TAX1BP1*^*−/−/−*^ cells compared with WT cells, whereas the reintroduction of TAX1BP1 attenuated this increase (Fig. 4b). In addition, we analyzed the expression levels of spliced and unspliced XBP1 mRNA. In *TAX1BP1*^*−/−/−*^ cells, we observed a significant increase in spliced XBP1 (XBP1sp) mRNA expression level compared with WT DT40 cells and *TAX1BP1*^*−/−/−*^*/Tax1bp1* cells (Fig. 4c). We next examined surface IgM expression. As expected, surface IgM expression was decreased in *TAX1BP1*^*−/−/−*^ cells compared with WT cells, and reintroduction of TAX1BP1 reconstituted surface IgM expression (Fig. 4d). We also confirmed the decreased surface IgM expression and induced XBP1 mRNA splicing (XBP1sp) in splenic B cells from *TAX1BP1*^*−/−*^ mice compared with WT mice (Supplemental Fig. S2b,c). These data suggest that TAX1BP1 disruption enhanced splicing of the membrane-type IgM heavy chain (μM) mRNA to the secretory type and enhanced the observed aberrant differentiation into antibody-secreting plasma cells. We further assessed the B cell development in *TAX1BP1*^*−/−*^ mice. *TAX1BP1*^*−/−*^ mice showed normal frequencies of pre-pro B cell, immature and recirculating B cells in the bone marrow (Supplemental Fig. S3a) and T1, T2, marginal and follicular B cells compared with WT mice (Supplemental Fig. S3b,c). In addition, total number of splenocytes and splenic B cells was not changed in *TAX1BP1*^*−/−*^ mice (Supplemental Fig. S3d).

### Splenic germinal center hypoplasia in TAX1BP1-deficient mice

**Figure 5:**
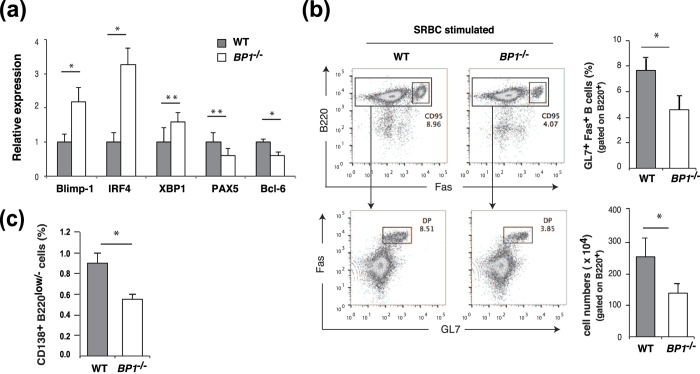
TAX1BP1 deficiency reduced splenic germinal center (GC) B cells in mice. WT and TAX1BP1-deficient mice were analyzed after secondary immunization with sheep red blood cells (SRBC). (**a**) Relative quantification of the levels of Blimp-1, IRF4, XBP1, PAX5, and Bcl-6 transcripts in sorted spleen B cells from SRBC-immunized WT and *TAX1BP1*^*−/−*^ (*BP1*^*−/−*^) mice. The transcript levels are relative to the transcript levels in splenic B cells from WT mice, which are set at 1. The results are shown as the means and s.e.m. (n = 5). *P < 0.01, **P < 0.05. P values are based on two-tailed Student t tests. (**b**) Representative flow cytometric plots of splenic GC B cells after secondary immunization with SRBCs; cells were stained for the B cell and GC markers B220 and Fas (top row) or Fas and GL7 (bottom row: gated on B220^+^ cells). The numbers adjacent to the gated area indicate the percentage of cells in the gate. The percentages and numbers of splenic GC B cells in WT and *TAX1BP1*^*−/−*^ (*BP1*^*−/−*^) mice were statistically analyzed. The results are shown as the means and standard deviation (n = 3). *P < 0.05. P values are based on two-tailed Student t tests. (**c**) The percentage of CD138^+^ B220^low/−^ cells in WT and *TAX1BP1*^*−/−*^ (*BP1*^*−/−*^) mice was statistically analyzed. The results are shown as the means and standard deviation (n = 3). *P < 0.05. P values are based on two-tailed Student’s t tests.

To study the physiological role of TAX1BP1 in B-lymphocyte activation and differentiation and GC formation, we immunized WT and *TAX1BP1*^*−/−*^mice intraperitoneally using sheep red blood cells (SRBCs), a potent T-cell-dependent antigen that induces GC formation. After the second immunization, we isolated splenic B cells from the immunized WT and *TAX1BP1*^*−/−*^ mice and measured the gene expression levels of transcription factors involved in GC formation. According to our quantitative PCR analysis, splenic B cells from SRBC-immunized *TAX1BP1*^*−/−*^ mice expressed much higher levels of Blimp-1 when compared with WT cells, similar to the previous findings in TAX1BP1-deficient DT40 cells (Fig. 5a). Xbp-1 expression, which is induced by Blimp-1, was also upregulated in *TAX1BP1*^*−/−*^ mice, whereas the expression of Pax5 and Bcl-6, which are repressed by Blimp-1, was downregulated in TAX1BP1-deficient splenic B cells (Fig. 5a).

Furthermore, we used flow cytometry to evaluate GC B cells in single-cell suspensions of splenic B cells isolated from SRBC-immunized WT and *TAX1BP1*^*−/−*^ mice. The GL7 monoclonal antibody reacts with a cell-surface protein found on activated GC B cells. Notably, the population of these splenic GC B cells coexpressing the GC markers GL7 and Fas^29^,^30^ was reduced in SRBC-immunized *TAX1BP1*^*−/−*^ mice compared with WT mice (Fig. 5b). We also used flow cytometry to compare the frequency of plasma cells and plasmablast cells, which express cell surface antigen CD138 (syndecan-1) and low levels of B220^31^,^32^, among splenic B cells isolated from WT and *TAX1BP1*^*−/−*^ mice after SRBC immunization. This analysis revealed that *TAX1BP1*^*−/−*^ mice exhibited profound reduction of plasma cells compared with WT mice after SRBC immunization (Fig. 5c).

**Figure 6:**
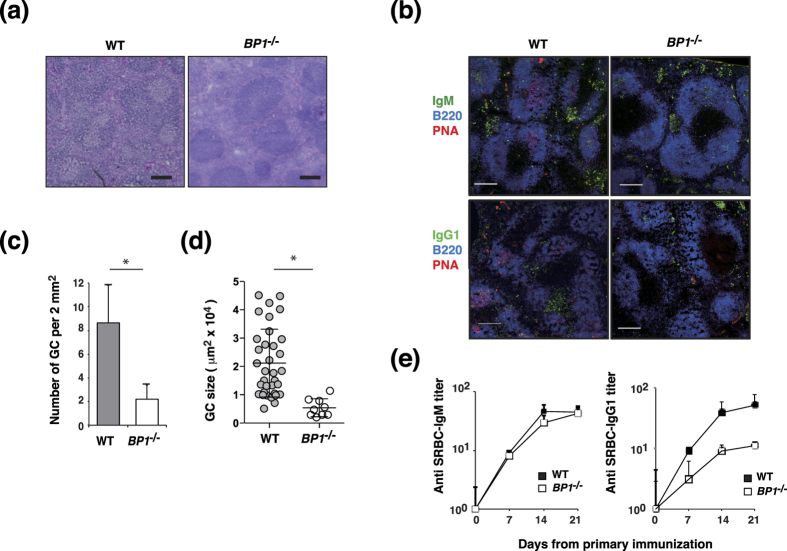
Histological analysis of splenic germinal center (GC) B cells. (**a**) Paraffin-embedded spleen sections from sheep red blood cell (SRBC)-immunized WT and *TAX1BP1*^*−/−*^ (*BP1*^*−/−*^) mice were stained with hematoxylin-eosin and visualized via light microscopy. The data are representative of 4 experiments. Scale bars: 200 μm. (**b**) Immunohistochemical analysis of GC B cells. Spleens from SRBC-immunized WT and *TAX1BP1*^*−/−*^ (*BP1*^*−/−*^) mice were visualized by staining with a monoclonal antibody against B220 as well as biotin-labeled PNA (GCs), IgM and IgG1-specific antibodies on day 10 after SRBC immunization. The data are representative of 4 experiments. Scale bars: 200 μm. (**c,d**) The number (**c**) and size (**d**) of GCs in spleen sections of immunized WT and *TAX1BP1*^*−/−*^ (*BP1*^*−/−*^) mice. Individual dots represent each GC. TAX1BP1 deficiency caused significant decreases in the number and size of GCs. The results are shown as the means and standard deviation (n = 4). (**c**) *P < 0.005. P values are based on two-tailed Student t tests. (**d**) *P < 0.0001. P values are based on Mann-Whitney U test. (**e**) Serum SRBC-specific IgM and IgG1 levels were determined via enzyme-linked immunosorbent assay with SRBC-coated plates (Life Diagnostics, Inc., West Chester, PA, USA) according to the manufacturer’s instructions. The results are shown as the means and standard deviation (n = 4).

This finding was further confirmed by a histological analysis of hematoxylin–eosin (H&E) stained splenic sections. Prominent GC formation was observed in the spleens of SRBC-immunized WT mice; in contrast, GC formation was impaired in the spleens of SRBC-immunized *TAX1BP1*^*−/−*^ mice (Fig. 6a). Examination of spleens by immunohistochemistry also showed that *TAX1BP1*^*−/−*^ mice displayed significant reductions in GC size and number compared with WT mice at day 10 after SRBC immunization (Fig. 6b–d). IgM^+^ plasma cells were similarly present in spleen sections of WT and *TAX1BP1*^*−/−*^ mice, however, IgG^+^ plasma cells were reduced in *TAX1BP1*^*−/−*^ mice (Fig. 6b). We next sought to determine whether the reduced GC formation and plasma cell differentiation in *TAX1BP1*^*−/−*^ mice would affect antigen-specific antibody production. Accordingly, we evaluated the serum levels of SRBC-specific IgM and IgG1 antibodies from WT and *TAX1BP1*^*−/−*^ mice following SRBC immunization. Both WT and *TAX1BP1*^*−/−*^ mice exhibited elevated serum SRBC-specific IgM (Fig. 6e) and IgG1 titers. However, *TAX1BP1*^*−/−*^ mice had lower SRBC-specific IgG1 titers compared with WT mice. These findings suggest that TAX1BP1 regulates both GC formation and specific antibody production.

## Discussion

On encountering antigen, B cells alter their physiological state and localization and initiate differentiation through a GC response. GCs are critical for the generation of memory B cells and plasma B cells that produce high-affinity antibodies; a process strictly regulated by specific transcription factors such as Bcl-6 and Blimp-1^2^,^33^.

Bcl-6-deficient mice exhibit defective T-cell-dependent immune responses as a result of completely abolished GC formation, whereas constitutive Bcl-6 expression inhibits B cell plasma cell differentiation^34^,^35^. B cells must switch off Bcl-6 expression and switch on transcription factors such as Blimp-1 to exit the GC and differentiate into plasma cells. Blimp-1 is required for plasma cell differentiation because it represses a large set of genes required for GC B-cell development and cell proliferation (e.g., Bcl-6) and executes a plasma cell-specific transcription program established by IRF4, Blimp-1, and X-box-binding protein 1 (XBP1)^8^,^36^,^37^. In response to NF-κB signaling, IRF4 expression directly activates Blimp-1 to promote plasma cell differentiation^38^,^39^ and counteract Bcl-6-mediated repression of the Blimp-1 promoter^40^. In addition, ERK activation is critical for inducing the expression of Blimp-1^26^. Recently, constitutive Blimp-1 activation was found to impair the differentiation of GC B cells in mice lacking Srg3, a component of the SWI/SNF complex that acts with Bcl-6 to repress the Blimp-1 promoter^41^. Taken together, these findings indicate that the regulation of Blimp-1 expression is critical for GC formation and plasma cell differentiation; however, the involvement of these intracellular signaling systems in Blimp-1 repression remains unknown.

TAX1BP1, a ubiquitin binding protein, functions as an adaptor to recruit its catalytic partner A20 to K-63 polyubiquitinated proteins such as TRAF6 and RIP1. This ubiquitin-editing enzyme combines with Itch and RNF11 to negatively regulate NF-κB signaling pathways^15^,^23^. TNF or interleukin 1 (IL-1)-induced TAX1BP1 phosphorylation by IKKα is pivotal to the assembly of this quadruple protein complex and termination of NF-κB signaling^42^. A20-deficient B cells exhibit enhanced NF-κB pathway activation in response to αIgM, LPS, and αCD40 stimulation; however, MAPK pathway activation and AKT phosphorylation remain unchanged^18^,^43^. Mice with B-cell-specific reduction of A20 expression harbor elevated numbers of GC B cells, autoantibodies, and glomerular immunoglobulin deposits^43^, and *A20*-deficient B cells are resistant to Fas-mediated cell death, likely because of the increased expression of NF-κB-dependent antiapoptotic proteins such as Bcl-x. These findings indicate that A20 restricts B cell survival, but it protects other cells from TNF-induced cell death. Despite studies demonstrating that A20 restricts autoimmune responses, it has remained unclear how A20 and its partner TAX1BP1 regulate B cells or plasma cell transcription factors.

To investigate the role of TAX1BP1 in B-cell activation and plasma cell differentiation, we generated a TAX1BP1-deficient line of DT40 B cells. The depletion of TAX1BP1 in DT40 cells caused a slight increase in NF-κB pathway activation in response to B cell activation via LPS and αCD40. TAX1BP1-deficient DT40 cells also showed an increased level of TRAF6 polyubiquitination, which contributes to CD40-mediated ERK activation^24^,^44^ (Supplementary Fig. S1). Thereby, ERK phosphorylation was significantly increased and prolonged in TAX1BP1-deficient DT40 cells and splenic B cells from *TAX1BP1*^*−/−*^ mice (Fig. 2) in contrast to *A20*-deficient B cells in which Erk phosphorylation was not altered^18^. TAX1BP1 restoration attenuated the constitutive ERK signaling observed in TAX1BP1-deficient cells (Fig. 3). TAX1BP1 may restrict CD40-induced ERK phosphorylation in an A20-independent pathway. It will be interesting to investigate whether A20 and TAX1BP1 signaling pathways can act independently to regulate ERK activity in GC formation and B-cell differentiation. In B cells, ERK pathway activation is critical for inducing Blimp-1^26^. TAX1BP1 disruption enhanced ERK activity and induced constitutively higher expression levels of Blimp-1, and treatment with the MEK inhibitor U0126 inhibited the expression of Blimp-1 (Fig. 3). Xbp1, which is induced by Blimp-1, was also strongly expressed, whereas the expression levels of Pax5 and Bcl-6, which are repressed by Blimp-1, were significantly reduced. We also confirmed that constitutive Blimp-1 expression in TAX1BP1-deficient DT40 B cells leads to spontaneous plasmacytic differentiation (Figs 3 and 4). TAX1BP1 may regulate the termination of αCD40-induced NF-κB and ERK signaling, thereby restricting the continuous expression of Blimp-1.

CD40 induction is a critical step in the T-cell-dependent immune response^45^,^46^ that contributes to GC formation, memory B-cell development, Ig isotype switching, and affinity maturation. Previous *in vivo* experiments have demonstrated that constitutively active CD40 receptors can prolong B-cell survival and increase proliferation; however, these B cells exhibit impaired GC formation^47^. To evaluate the physiological role of TAX1BP1 in the adaptive immune response, we immunized WT and *TAX1BP1*^*−/−*^mice intraperitoneally with SRBCs. The splenic B cells from SRBC-immunizes *TAX1BP1*^*−/−*^ mice showed high expression of Blimp-1 and downregulation of Bcl-6, a critical regulator of the GC reaction, similar to TAX1BP1-deficient DT40 cells (Fig. 5a). Despite the induction of a strong GC reaction in WT mice, *TAX1BP1*^*−/−*^ mice exhibited impaired GC formation, resulting in the attenuation of plasma cell and antigen-specific antibody production (Figs 5 and 6). These results suggest that Blimp-1 activation induced aberrant plasma cell differentiation and downregulation of Bcl-6 expression impaired the germinal center formation and then leading significant reduction of plasma cells in *TAX1BP1*^*−/−*^ mice compared with WT mice after SRBC immunization (Fig. 5c).

Our results demonstrate that in B cells, TAX1BP1 restricts CD40-mediated responses and terminates NF-κB and ERK signaling to restrict aberrant Blimp-1 expression, regulate plasma cell development, and initiate GC formation. Accordingly, TAX1BP1 appears to be a key regulator of GC reactions.

## Methods

### Cells and media

WT and its derivative mutant DT40 cells were cultured in RPMI1640 medium supplemented with 10% fetal calf serum (FCS), 1% chicken serum, 50 μM 2-mercaptoethanol, 2 mM L-glutamine, and antibiotics.

### Generation of TAX1BP1 deficient DT40 cells (Bsr/Ecogpt/His)

The *Tax1bp1* gene was disrupted by gene targeting. The targeting constructs were designed to replace the first exon with the HisD, Bsr or Ecogpt resistance gene cassette. The gene disruption is expected to remove the genomic region that encodes chicken TAX1BP1 amino acids 1 to 81. The linearized targeting vector was introduced into WT DT40 cells. Transfections by electroporation and selections of the clones were done as previously described^48^. The disruption of *Tax1bp1* gene was screened by genomic southern blotting using probe indicated in Fig. 1 and genome DNA digested by HindIII. The TAX1BP1 probe was amplified by PCR using chicken genomic DNA as a template with the following primers: (forward: 5′-CATGATCCTGTGATTCGATGACGTGC-3′, reverse: 5′-GATGCTACTTTATGGAATACGCTGGGAG-3′). All the methods involved in DNA recombination were approved by Committees of Tokyo University of Pharmacy and Life Sciences (Tokyo, Japan) and carried out in accordance with approved University guidelines.

### Generation of TAX1BP1^*−/−/−*^/Tax1bp1 cells

The coding sequence of chicken *TAX1BP1* was amplified from DT40 cDNA with the primers (forward: 5′-CGGCTCGAGGTATGTCATCCTTTCAAG-3′, reverse: 5′-CGGCTCGAGTTAATCAAAGTTTAAAA-3′). PCR products were cloned into pcDNA3.1/Zeo and the insert was sequenced. The chicken TAX1BP1 expression vector was transfected in *TAX1BP1*^*−/−/−*^ cells, and transfectant cells were selected in the presence of Zeocin (1 mg/ml). *Tax1bp1* gene stable expression was verified.

### Mice and immunization

Establishment of *TAX1BP1*^*−/−*^ mice was described previously^15^. For analysis of GC formation and antibody responses, 8- to 10-week-old male *TAX1BP1*^*−/−*^ mice and their WT littermates were immunized intraperitoneally with sheep red blood cells (SRBC) (10^8^ cells/mouse) (Nippon Bio-Test Laboratories inc.). Secondary challenges were done 1 week after primary immunization and spleens were collected for analysis at 10 days after primary immunization. All procedures involving mice were approved by the Laboratory Animal Care and Committees of Tokyo University of Pharmacy and Life Sciences (Tokyo, Japan). All mice were maintained under specific pathogen–free conditions. All the methods were carried out in accordance with the Guidelines for Animal Experiments of Tokyo University of Pharmacy and Life Sciences (Tokyo, Japan).

### Magnetic cell sorting and flow cytometry

RBC-depleted spleen cell suspensions prepared from WT and *TAX1BP1*^*−/−*^mouse were labeled with CD11b and CD90.2 microbeads (Miltenyl Biotec). Then the B cells were enriched by negative selection using autoMACSpro separator (Miltenyl Biotec) according to the manufacturer’s instructions. The collected cells were stained with the following antibodies: APC anti-B220, PE anti-Fas, PE anti-CD138 (Syndecan-1), FITC anti mouse IgM, FITC anti-GL7 antigen (BioLegend) and 7-AAD (BioLegend). Single-cell suspensions of bone marrow from WT and *TAX1BP1*^*−/−*^ mouse were stained with following antibodies: PE/Cy7 anti-TCR β chain, 7-AAD, PerCP-Cy5.5 anti-Gr-1, APC anti-B220, and FITC anti-mouse IgM (BioLegend). Single-cell suspensions of total splenocytes from WT and *TAX1BP1*^*−/−*^ mouse were stained with different combinations of the following antibodies: PE/Cy7 anti-TCR β chain, PerCP-Cy5.5 anti-Gr-1, 7-AAD, FITC anti-mouse IgM, APC anti-CD23, PE anti-CD21 (BioLegend), or PerCP-Cy5.5 anti-Gr-1, 7-AAD, PE/Cy7 anti-B220, PE anti-CD93, FITC anti-mouse IgM and APC-CD23 (BioLegend). WT DT40 cells, *TAX1BP1*^*−/−/−*^ cells and *TAX1BP1*^*−/−/−*^*/Tax1bp1* cells were stained with FITC conjugated anti chicken IgM (Bethyl). FACS Verse (BD) and SH800 (Sony Biotechnology Inc.) were used for flow cytometry, and results were analyzed with FlowJo software.

### Reverse transcription PCR and XBP1 splicing assay

The disruption of *Tax1bp1* gene in TAX1BP1 deficient DT40 cells was confirmed by amplifying coding region of *Tax1bp1* gene by reverse transcriptase PCR. Total RNA was isolated using ReliaPrep RNA cell miniprep system (Promega), and converted to cDNA by reverse transcriptase (TOYOBO). A part of chicken TAX1BP1 was amplified with KOD plus DNA polymerase (TOYOBO). A PCR reaction with chicken *Gapdh* specific primers were used as a positive control. The following Primers were used: chicken *TAX1BP1* (forward: 5′-ACTTGGCAAGACAGCAGGAAATCAAGCTGG-3′, reverse: 5′-GGCTGACTGCACACTACATCATCTTCTAGC-3′), chicken *β-Actin* (forward: 5′-GACATGGAGAAGATCTGGCA-3′, reverse: 5′-GGTCTCAAACATGATCTGGGT-3′).

TAX1BP1: 1842-1872, 2082-2111 (270bp)

β-actin: 252-271, 369-389 (138 bp)

The XBP1-splicing assay was done as previously described^49^. XBP1 expression of wild-type DT40, *TAX1BP1*^*−/−/−*^*, TAX1BP1*^*−/−/−*^*/Tax1bp1,* cell line and isolated splenic B cells from unimmunized WT and *TAX1BP1*^*−/−*^ mice was analyzed by RT-PCR. Primers encompassing the spliced sequences were used for PCR amplification with KOD plus DNA polymerase (TOYOBO), and products were separated by electrophoresis through a 2.5% agarose gel and visualized by ethidium bromide staining. A PCR reaction with *β-Actin* specific primers were used as a positive control. The following Primers were used: chicken XBP1 (forward: 5′-GTGCGAGTCTACGGATGTGA-3′, reverse: 5′-AAGCCGAACAGGAGATCAGA-3′). chicken β*-Actin* (forward: 5′-GATATGGAGAAGATCTGGCA-3′, reverse: 5′-GGTCTCAAACATGATCTGTGT-3′), mouse *XBP1* (forward: 5′-ACACGCTTGGGAATGGACAC-3′, reverse: 5′-CCATGGGAAGATGTTCTGGG-3′), mouse β*-Actin* (forward: 5′-GACATGGAGAAGATCTGGCA-3′, reverse: 5′-GGTCTCAAACATGATCTGGGT-3′).

### Luciferase assays

Cells were transfected with an NF-κB luciferase reporter (Clontech) and the Renilla reporter pRL-CMV (Promega) as an internal control. Cells were harvested 24 h after transfection and incubated with or without indicated reagents, 2.5 μg/ml anti-CD40 antibody (αCD40) (AbD Serotec) and 50 ng/ml LPS (Sigma) for 8–24 h, and cell lysates were analyzed by dual-luciferase assay according to the manufacturer’s instructions (Promega). Results presented are the average of triplicate experiments with the S.D. values shown as error bars.

### Co-immunoprecipitation and immunoblotting

WT DT40 cells and *TAX1BP1*^*−/−/−*^ cells were incubated for indicated time after CD40 activation and lysed in RIPA buffer. Lysates were incubated with anti-TRAF6 antibody (abcam) or relative non-immunized IgG for 3 h at 4 °C followed by protein G Sepharose for another 2 h. Proteins were detected by anti-TRAF6 (Santa Cruz Biotechnology), anti-β-actin (SIGMA) and anti-K63 Ubiquitin (Millipore).

Whole-cell lysates from DT40 cells were separated by SDS-PAGE gels and transferred to Immobilon-P membrane (Millipore). The membranes were blotted with the following antibodies: I-κBα, Phospho-I-κBα, JNK, Phospho-JNK, p44/42 MAPK, phospho-p44/42 MAPK (Cell Signaling), TAX1BP1 (Abnova), β-actin (SIGMA).

### Enzyme-linked immunosorbent assay (ELISA)

Immunoglobulin concentrations in the serum and cell medium were measured by standard sandwich ELISA. Concentrations of antibodies specific for SRBCs were determined by ELISA using the mouse anti-SRBC ELISA kit (Life Diagnostics, Inc.), according to the manufacturer’s instructions. Concentrations of chicken IgM were determined by ELISA using chicken IgM ElISA kit (Bethyl Laboratories, Inc), according to the manufacturer’s instructions.

### Real-time PCR

Total RNA was extracted from the DT40 cell lines and isolated splenic B cells from unimmunized or SRBC-immunized mice using ReliaPrep RNA Cell Miniprep System (Promega) and used as a template to create cDNA by ReverTra Ace qPCR RT kit (TOYOBO). Quantitative PCR analysis was performed using Thunderbird SYBR qPCR Mix (TOYOBO) in a Rotor-Gene Q (Qiagen) according to the manufacturer’s instructions. The following Primers were used:

chicken *Bcl-6* (forward: 5′-TGGAGCACGTGGTTGATACT-3′, reverse: 5′-ACAGCAGACACCATCTCAGC-3′), chicken *Prdm1/Blimp1* (forward: 5′-ACACAGCGGAGAGAGACCAT-3′, reverse: 5′-GCACAGCTTGCACTGGTAAG-3′), chicken *IRF4* (forward: 5′-AGCGCAACTGGAGAGAATTT-3′, reverse: 5′-TCCTGTCACCTGACAACCAT-3′), chicken *Pax5* (forward: 5′-GTCAGCCACGGCTGCGTCAGCAAAATAC-3′, reverse: 5′-GGCTGCTGCACCTTTGTCCGTATGAT-3′), chicken *XBP1* (forward: 5′-GTGCGAGTCTACGGATGTGA-3′, reverse: 5′-AAGCCGAACAGGAGATCAGA-3′), chicken μ*S* (forward: 5′-GGAGAACCCCGAAAATGAGT-3′, reverse: 5′-GCCAACACCAAGGAGACATT-3′), chicken μ*M* (forward: 5′-GGAGAACCCCGAAAATGAGT-3′, reverse: 5′-GTTGGATGTCGTCGTCCTCT-3′), chicken *GAPDH* (forward: 5′-CCATCACAGCCACACAGAAGA-3′, reverse: 5′-CTTTCCCCACAGCCTTAGCA-3′), mouse *Prdm1/Blimp1* (forward: 5′-CCAGGTCTGCCACAAGAGATT-3′, reverse: 5′-TCCGATGACTCATAGAGGCTG-3′), mouse *Bcl-6* (forward: 5′-CCAACCTGAAGACCCACACTC-3′, reverse: 5′-GCGCAGATGGCTCTTCAGAGTC-3′), mouse *IRF4* (forward: 5′-ATGAACTTGGAGACGGGCAGCCGGGGCTC-3′, reverse: 5′-CTGGCTTGTCGATCCCTTCTCGGAACTT-3′), mouse *PAX5* (forward: 5′-GATGTAGTCCGCCAAAGGAT-3′, reverse: 5′-GCTTGATGCTTCCTGTCTCA-3′), mouse *XBP1* (forward: 5′-GTGGATTTGGAAGAAGAGAAC-3′, reverse: 5′-AGAGAAAGGGAGGCTGGTAAG-3′), mouse *GAPDH* (forward: 5′-ATGGTGAAGGTCGGTGTGAACGGATT-3′, reverse: 5′-AGCTTCCCATTCTCGGCCTTGACTG-3′). MEK inhibitor U0126 was from Cell Signaling. DMSO was from Nacalai tesque. The relative amounts of mRNA were normalized using glyceraldehyde 3-phosphate dehydrogenase mRNA as an invariant control.

### Histological and immunohistochemical analysis

For histological analysis, mouse spleen were fixed in 10% formalin, dehydrated in 100% ethanol, and embedded in paraffin wax at 58 °C. For H&E staining, sections (6 μm) were rehydrated and stained with hematoxylin and eosin. For immunohistochemical analysis, frozen 6 μm sections were thawed, air-dried, methanol-fixed, and stained for 1 hour at room temperature in a humidified chamber with anti-mouse IgM-FITC, anti-mouse IgG1-FITC (Santa Cruz Biotechnology), anti-mouse B220 (Biolegend) and biotinylated peanut agglutinin (VECTOR laboratories) followed by Cy3-streptavidin (BioLegend) and Alexa Fluor 405 goat anti-rabbit IgG (Molecular Probes). Imaging was performed using a fluorescence microscopy (BZ-9000, Keyence) and a confocal laser scanning microscopy (FV1000-D, Olympus). Immunohistochemical images of the spleen samples were used to determine frequency of GC per 2 mm^2^. On these images, every germinal center (PNA-positive) was identified manually and measured in ImageJ to calculate their size.

### Statistical analysis

Results were statistically compared using Student’s t-test or Mann-Whitney U test (for two group comparisons) and a one-way ANOVA (for three group comparisons). P-value of P < 0.05 was considered significant.

## Additional Information

**How to cite this article**: Matsushita, N. *et al*. Regulation of B cell differentiation by the ubiquitin-binding protein TAX1BP1. *Sci. Rep.* **6**, 31266; doi: 10.1038/srep31266 (2016).

## Supplementary Material

Supplementary Information

Supplementary Figure 1

Supplementary Figure 2

Supplementary Figure 3
