# Supplementary material for: Regulation of B cell differentiation by the ubiquitin-binding protein TAX1BP1
Source: Sci Rep. 2016 Aug 12;6:31266. doi: 10.1038/srep31266 (PMC4981851; doi:10.1038/srep31266)
Supplement: Supplementary Information [file srep31266-s1.pdf]

Supplementary Information

Regulation of B cell differentiation by the ubiquitin-binding protein TAX1BP1

Nobuko Matsushita*, Midori Suzuki, Emi Ikebe, Shun Nagashima, Ryoko Inatome, Kenichi Asano, Masato Tanaka, Masayuki Matsushita, Eisaku Kondo, Hidekatsu Iha and Shigeru Yanagi

*Email: matsun@toyaku.ac.jp

Supplementary Figure S1

Negative regulation of TRAF6 polyubiquitination by TAX1BP1

Wild-type (WT) DT40 cells and TAX1BP1-deficient (*TAX1BP1*$^{-/-/-}$) cells were stimulated with or without αCD40 for the indicated time. Proteins from lysates were immunoprecipitated with an antibody to TRAF6, eluted with 1% SDS, followed by immunoblotting with a K63-specific antibody to Ub or an antibody to TRAF6. Lysates were subjected to immunoblotting with antibodies to TRAF6 or β-actin. As a control, immunoprecipitates obtained with a relative non-immunized IgG incubated with non-treated cell lysate were used.

Supplement Figure S2

TAX1BP1 deficiency stimulates IgM secretion and XBP1 splicing

(a) Concentration of chicken IgM secreted in culture medium determined by quantitative ELISA. The cell density at the start of cultures of WT DT40, *TAX1BP1*$^{-/-/-}$ (*BP1*$^{-/-/-}$) and *TAX1BP1*$^{-/-/-}$*/Tax1bp1* (*BP1*$^{-/-/-}$*/Tax1bp1*) cells was 1x10^6 cells/ml, and culture medium samples were taken after 16 h. Culture medium chicken IgM levels were determined via enzyme-linked immunosorbent assay with chicken IgM-coated plates (Life Diagnostics, Inc., West Chester, PA, USA) according to the manufacturer's instructions. The graphs show the average and standard deviation of the chicken IgM concentration calculated from four independent cultures. *P < 0.05. P values are based on two-tailed Student t tests. **(b)** RT-PCR analysis of XBP-1 splicing in isolated splenic B cells in wild-type (WT) and *TAX1BP1*$^{-/-}$ (*BP1*$^{-/-}$) mice. **(c)** Surface immunoglobulin (sIgM) expression in splenic B cells from WT and *BP1*$^{-/-}$mice was analyzed by flow cytometry. Histograms indicate the relative cell number and logarithmic fluorescence intensity.

Supplementary Figure S3

Analysis of B cell development in WT and *TAX1BP1*$^{-/-}$ mice. Flow cytometry analysis of bone marrow (BM) and splenic B cell development in wild type and *TAX1BP1*$^{-/-}$ mice. The results are statistically analyzed and shown as the means and standard deviation (n=3).

(a) BM B cells from WT and *TAX1BP1*$^{-/-}$ mice were gated on 7AAD^{-} TCR β^{-} Gr-1^{-} cells and labeled with antibodies to B220 and IgM to enumerate pre-pro B cell (B220^{lo}, IgM^{-}), immature (B220^{lo}, IgM^{+}) and recirculating (B220^{hi}, IgM^{+}) B cells. Frequencies of pre-pro B cell, immature and recirculating B cell populations are shown as representative FACS images. The numbers adjacent to the gated area indicate the percentage of cells in the gate. **(b)** Spleen B cells from WT and *TAX1BP1*$^{-/-}$ mice were gated on 7AAD^{-} TCR β^{-} Gr-1^{-} IgM^{+} cells and labeled with antibodies to CD21 and CD23 to enumerate follicular (CD21^{lo}, CD23^{hi}) and marginal zone (CD21^{hi}, CD23^{lo}) B cells. Frequencies of follicular and marginal zone B cell populations are shown as representative FACS images. **(c)** Spleen B cells from WT and *TAX1BP1*$^{-/-}$ mice were gated on 7AAD^{-} Gr-1^{-} B220^{+} CD93^{hi} cells and labeled with antibodies to CD23 and IgM to enumerate T1 (CD23^{lo}, IgM^{+}), T2 (CD23^{hi}, IgM^{+}), T3 (CD23^{hi}, IgM^{-}) B cells. Representative FACS plots of T1, T2, T3 B cells in spleen of WT and *TAX1BP1*$^{-/-}$ (*BP1*$^{-/-}$) mice. **(d)** Total number of splenocytes and total number of B cells in the spleen of WT and *TAX1BP1*$^{-/-}$ (*BP1*$^{-/-}$) mice.
