# Supplementary figures and images for: Regulation of B cell differentiation by the ubiquitin-binding protein TAX1BP1
Source: Sci Rep. 2016 Aug 12;6:31266. doi: 10.1038/srep31266 (PMC4981851; doi:10.1038/srep31266)

Figure S1

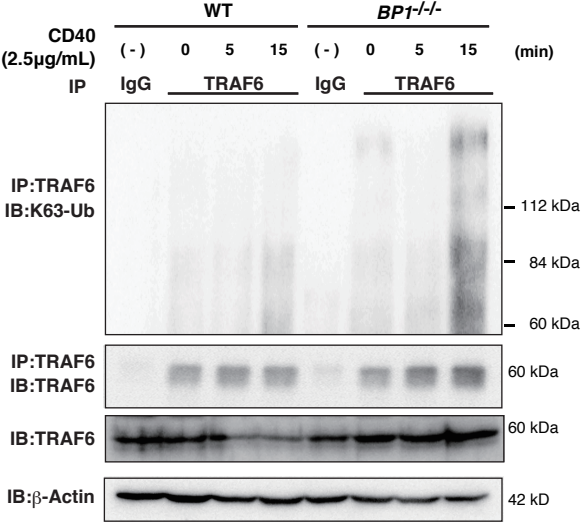

Supplement: Supplementary Figure 1 [file srep31266-s2.pdf]

Figure S2

(a)

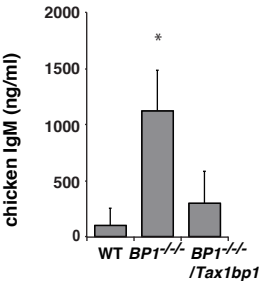

(b)

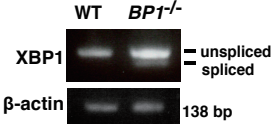

(c)

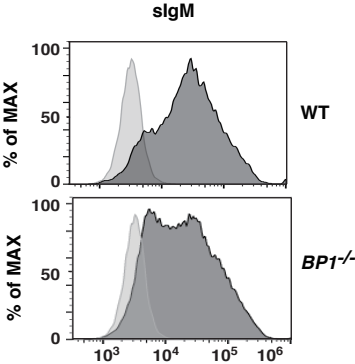

Supplement: Supplementary Figure 2 [file srep31266-s3.pdf]

**Figure S3****(a)**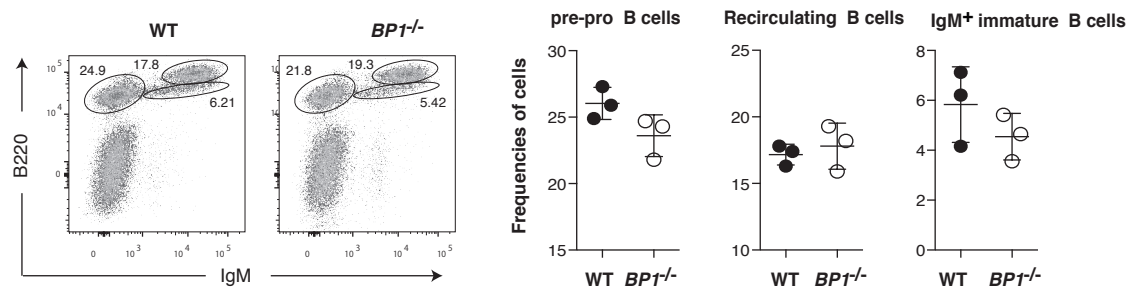**(b)**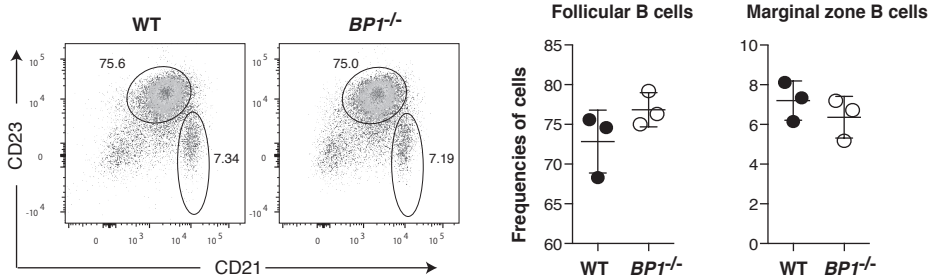**(c)**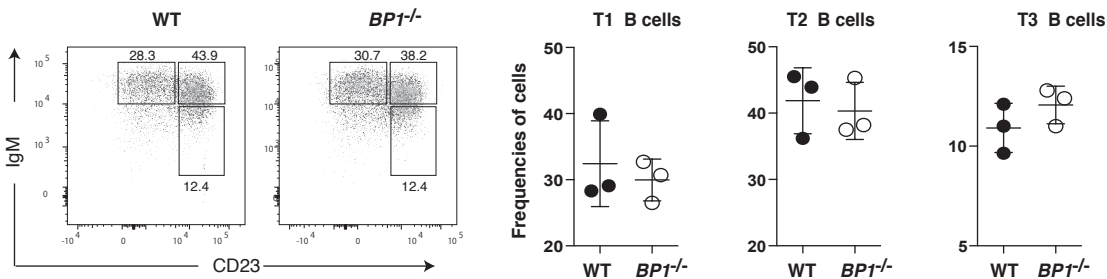**(d)**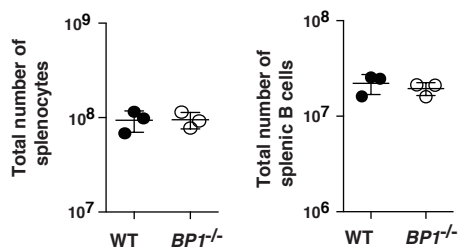

Supplement: Supplementary Figure 3 [file srep31266-s4.pdf]
